# Optimising Referrals: A Closed Loop Quality Improvement on Blood Test Compliance in Memory Clinic Referrals

**DOI:** 10.1192/bjo.2026.11401

**Published:** 2026-06-30

**Authors:** Naduni Jayasinghe, Thomas Rose, Claire Weaver, Malarvizhi Sandilyan

**Affiliations:** Prospect Park Hospital, Reading, United Kingdom

## Abstract

**Aims:**

Dementia commonly affects older adults, with over 944,000 affected and one in eleven adults aged over 65 in the UK diagnosed. Due to the increasing number of referrals to memory clinics (MC), the recognition and management of reversible causes of cognitive impairment in primary care is essential. During the first cycle, missing information and blood tests were found to delay patient care and reduce efficiency. The National Institute for Health and Care Excellence (NICE) recommends the following: full blood count (FBC), urea and electrolytes (U&Es), liver function tests (LFTs), thyroid function tests (TFTs), glycated haemoglobin (Hbs-352c), serum calcium (Ca), erythrocyte sedimentation rate (ESR) or C-reactive protein (CRP), and serum vitamin B12 and folate (B12/folate). The second cycle assessed whether a specialised proforma and education leaflets for primary care clinicians increased the availability of blood tests within two months of referral.

**Methods:**

Patient information was extracted from referrals received over two twelve-week time periods, one and a half years apart. Cycle one was conducted between 10/10/2023 and 20/11/2023, and cycle two between 07/08/2025 and 28/10/2025. Data on referrals and blood tests, completed within two months of referral, were obtained from RiO and ICE West and analysed in Excel, with data anonymised. Patients with an existing dementia diagnosis or those referred directly by secondary care were excluded. After the first cycle, focus groups were held with the multi-disciplinary MC team, followed by the distribution of an information update letter and updated proforma to all primary care clinicians.

**Results:**

A total of 101 referrals were included. In Cycle one, only 11.9% of patient referrals had all recommended blood tests completed within two months prior to referral. 27.1% hadnone of the recommended tests. In Cycle two, 85.7% of referrals used the distributed proforma. The proportion of patients with all recommended blood tests completed increased to 23.8%. Of the referrals utilising the proforma, 80% included all relevant blood tests two months prior to the referral receival.

**Conclusion:**

Overall, 85.7% of referrals used the MC proforma. Using the proforma improved referral quality by prompting inclusion of relevant information and blood tests, enhancing service efficiency. Notably, around 75% of referrals lacked vital blood tests, affecting the efficiency of MC services. Focus group discussions highlighted that MC staff should now refer referrals lacking up-to-date bloods back to primary care and that wider distribution of the proforma with clear blood test requirements may further increase compliance, suggesting a further audit cycle could be beneficial in future.

